# Optimization of Process Parameters for Additively Produced Tool Steel 1.2709 with a Layer Thickness of 100 μm

**DOI:** 10.3390/ma14112852

**Published:** 2021-05-26

**Authors:** Vladislav Andronov, Jan Šimota, Libor Beránek, Jiří Blažek, Filip Rušar

**Affiliations:** 1 Department of Machining, Process Planning and Metrology, Faculty of Mechanical Engineering, Center of Advanced Aerospace Technology, The Czech Technical University in Prague, 160 00 Prague, Czech Republic; jan.simota@fs.cvut.cz (J.Š.); libor.beranek@fs.cvut.cz (L.B.); jiri.blazek@fs.cvut.cz (J.B.); 2 Additive Technology Department, CARDAM s.r.o., 252 41 Dolní Břežany, Czech Republic; filip.rusar@cardam-solution.cz

**Keywords:** Direct Metal Laser Sintering (DMLS), 3D printing, additive manufacturing, layer thickness, energy density, 3D printing parameters optimization

## Abstract

The purpose of this study was to find and optimize the process parameters of producing tool steel 1.2709 at a layer thickness of 100 μm by DMLS (Direct Metal Laser Sintering). HPDC (High Pressure Die Casting) tools are printed from this material. To date, only layer thicknesses of 20–50 μm are used, and parameters for 100 µm were an undescribed area, according to the state of the art. Increasing the layer thickness could lead to time reduction and higher economic efficiency. The study methodology was divided into several steps. The first step was the research of the single-track 3D printing parameters for the subsequent development of a more accurate description of process parameters. Then, in the second step, volume samples were produced in two campaigns, whose porosity was evaluated by metallographic and CT (computed tomography) analysis. The main requirement for the process parameters was a relative density of the printed material of at least 99.9%, which was achieved and confirmed using the parameters for the production of the samples for the tensile test. Therefore, the results of this article could serve as a methodological procedure for optimizing the parameters to streamline the 3D printing process, and the developed parameters may be used for the productive and quality 3D printing of 1.2709 tool steel.

## 1. Introduction

Additive manufacturing technologies create a wide range of possibilities that, when using conventional methods, are unattainable or financially unacceptable. Thanks to their flexibility, it is possible to reduce the time from design to the final product to a minimum and produce seemingly non-manufacturable components. With their constant development, their wider and more efficient use across a range of different applications occurs, they save money and time, but they also reduce the burden on the environment. The principle of metal additive manufacturing technology is based on the gradual formation of layers on the construction platform. The layer thickness of the applied material has some effect on the financial demands of the overall production process. The layer thickness in standard applications was in range 20−50 μm. By increasing the layer thickness to 100 μm, an increase in productivity may be achieved, which could lead to considerable financial savings [1,2,3].

In the beginning, the variation of combinations of individual parameters affects, among other things, the final quality of the product, the cost of production, and the duration of 3D printing. The improper combination can lead to unwanted porosity, print instability, and a negative change in mechanical properties. The main parameters that can be commonly varied include layer thickness (t), scanning speed (v), laser power (P), and hatch spacing (h) [4]. The dependence of these parameters is shown by the energy density, which belongs to the combined process parameters. It is given by a combination of individual input values, while its undeniable advantage is in the simple comparability of differently set processes. Linear, area, and volume energy density are commonly used (Equations (1)–(3)) [3].

(1) $$E_{1}=\frac{P}{v}\;[\frac{J}{\mathrm{mm}}],\;$$

(2) $$E_{2}=\frac{P}{v\cdot t}\;[\frac{J}{{\mathrm{mm}}^{2}}],$$

(3) $$E_{3}=\frac{P}{v\cdot h\cdot t}\;[\frac{J}{{\mathrm{mm}}^{3}}]$$

The individual quantities and their units are described above. The usual value of volume energy density for steel processing is in the range of 50–100 J/mm^3^, but it is possible to achieve very different values [3]. The effect of these key parameters has also been described in studies [3,5]. As a result of the choice of process parameters, the parameters that the authors monitor for the evaluation of the quality of the given component are influenced. Common product requirements include good metallurgical bonding, material homogeneity, mechanical properties, or high relative density. The higher the relative density of the material, the less it contains pores and is more suitable (unless it is a special application where porosity is desired). Relative density requirements are usually at least 99%. Hitzler et al.’s [6] review was excellent for finding more studies on this topic.

The influence of laser power and scanning speed on the relative density is discussed by Karg et al. [7], where 83 volume samples with different combinations of parameters were printed to obtain the final map. Schöber et al. [8] described the experimental determination of parameters for the 3D printing of material 1.2709 using single tracks, while the experiment was performed on a layer with a thickness of 100 μm. The laser power of 125 and 250 W was used to determine the ideal parameters. For the power of 125 W, the scanning speed was set in the range of 25−250 mm/s. For 250 W, the scan speed was 50 to 500 mm/s. The samples were first evaluated macroscopically by looking from the top when their continuity was evaluated. Subsequently, they were examined in a cross-section using metallographic sections. Based on these observations, areas of input parameters were marked during which no undesirable defects occur. During another experiment in the study [8], the hatch distance of the individual tracks h was investigated, which was set at 0.15 mm. At a value of 0.175 mm, a porous and coarse structure was formed, while at a value of 0.125 mm a repeated melting and spherical formation occurred. Król et al. [9] examined material 1.2709 based on varying laser speed, hatch distance, and layer thickness, while the last parameter was either 30 or 50 µm. The best relative density, approximately 99.3%, was found in a manufactured sample with 30 µm layer thickness, 340 mm/s laser speed, and 120 µm of hatch distance. Mugwagwa et al. [10] experimented with an M2 Cusing Concept Laser and an EOSINT M280 3D printing machine while changing the basic parameters, and it was found that both layer thicknesses yielded comparable relative densities at 180 W and 600 mm/s, which is 99.6% and 99.4%, respectively, for 30 and 45 µm layer thicknesses. Likewise, the mean relative densities of the 30 and 45 µm layer thicknesses were also quite similar (approximately 98.5%) at 180 W and 500 mm/s. As a result, the authors state that increasing the layer thickness results in a decline in both residual stresses and distortions, although an accompanying increase in unwanted porosity is also observed. Jarfors et al. [11] focused on the impact fracture strength and crack propagation characteristics of maraging steel 1.2709. In this study, the authors changed the build strategy (stripes, chessboard, and hexagon), scan speed, and hatching distance. The result was that for all strategies, the influence of the process parameters was similar where a greater hatch spacing promoted impact strength, and a greater interlayer rotation decreased the impact strength. De Souza et al. [12] evaluated the effect of high laser power (400 W), scan speed, layer thickness, porosity, and build direction on the microstructure and mechanical properties of maraging steel 300 parts built by SLM. One of the main findings of the authors was that the layer thickness was found to have a bigger influence on the manufacturing time than scan speed. Increasing the layer thickness by a factor of ~1.6 reduced the manufacturing time by up to 40% while keeping a reasonable surface roughness. Low porosity of 0.07% was achieved for samples requiring 39.6 min to be produced by SLM. Reducing the manufacturing time by ~30% increased the part porosity by ~0.23%. This increase in the porosity also reduced the yield stress by up to 30%. Hatos et al. [13] investigated the effect of skip melting of the layers during the 3D printing process. This was investigated by producing samples of 1.2709 steel (MS1) with an increased thickness of the melted layers. The starting layer thickness was 20 μm, which was increased in steps up to 160 μm with a 0.5 mm offset between the increased thickness layers. It has been concluded that skipping one or two layers does not cause a measurable increase in porosity while skipping more layers and melting a 120 μm thick layer, the input heat energy cannot melt the metal powder, significant porosity remains. The porosity increased exponentially by increasing the melted layer thickness. Kempen et al. [14] tested this on samples produced by SLM technology. Samples were built with a set of processing parameters chosen in terms of maximum density with a layer thickness of 30 μm, a scan speed of 150 mm/s, and a scan spacing of 112 μm (62% of the spot size). It was found that higher layer thickness and/or scan speed causes a decrease in density, which leads to a decrease in macro hardness. Moreover, there was no significant influence of layer thickness and scan speed on the microhardness of 1.2709 steel in the tested ranges. Suzuki et al. [15] examined the effects of laser power and scan speed on the relative density, melt pool depth, and Vickers hardness of selectively laser melted (SLM) maraging steel. These ranges were used in this study, P = 43–255 W and v = 417−3000 mm/s.

Narvan et al. [16] thoroughly investigated the additively produced material H13 on samples with a size of 10 × 10 × 15 mm^3^, which were made by SLM, manufactured using a laser power of 100, 200, and 300 W; scanning speed of 200, 400, 600, 800, 1000, and 1200 mm/s; and hatch spacing of 80 and 120 µm. A constant layer thickness of 40 µm, 67° scanning rotation between subsequent layers, and a stripe scanning strategy were maintained during the process. A preheating process of 200 °C was considered, and the best-achieved result of surface roughness 6.1 µm was achieved with P = 200 W, v = 600 mm/s, and h = 80 µm. Furthermore, this study states the significant influence of the preheating process of 200 °C in the production of the material H13. Wüst et al. [17] optimized the process parameters for SLM technology concerning surface quality. By choosing the optimal parameters, it was possible to reduce the roughness of the skin surfaces to Sa = 9.0 μm, which corresponds to a reduction of 40% compared to the manufacturer’s recommendations. The roughness of the contour surfaces could be reduced by 37.5% at Sa = 7.5 μm. Bhardwaj and Shukla [18] experimented with the parameters described below and different scanning strategies. The tests were performed by depositing single tracks by varying the laser power in the range of 250–350 W, scan speed in the range of 750–1250 mm/s, and hatch spacing (distance) in the range of 0.075–0.125 mm, keeping the layer thickness constant at 40 μm. The direction of the raster scan showed a strong influence on the texture and mechanical properties. This article also shows that the control of preferential columnar grain growth depends on the adopted scan strategy. Yuchao et al. [19] found that the relative density increases first and then decreases with the laser power, scanning speed, and scanning space. This is because a low laser power and a high scanning speed lead to low energy density, which is insufficient to melt the metal powder. A high energy density with high laser power and low scanning speed will bring strong vaporization and spatter, which leads to voids and inclusions. Local energy density will also increase with a small scanning space leading to voids and inclusions. A big scanning space will leave some powders in an unmelted state, which also leads to a low relative density. The orthogonal experiment in this study was carried out to find the optimized process parameters with a relative density higher than 99%. Letenneur et al. [20] used a set of density calibration artifacts built with laser power values from 160 to 350 W; the scanning speed from 500 to 2800 mm/s; the hatching space from 30 to 550 μm, and the layer thickness from 30 to 60 μm. The model was adapted for the IN625 alloy powder and an M280 EOS system. As it was widely assumed that the smaller the layer thickness, the better the surface finish and part precision, but the lower the build rate. The authors of this paper were recommended to work with layer thicknesses of 30 or 40 μm when precision is required and of 50 or 60 μm when process productivity is more important. Hatos et al. [21] examined the consequences of the production process occasionally needing to be interrupted due to lens cleaning, powder refilling, or technical problems. It was found that an increase in the layer thickness decreases with the elongation of a break, while strength values are not affected (lower than 160 μm). Energy absorbed by the samples during impact testing and different layer thickness shows a linear behavior as the break extends. The authors also stated that the sample that contains layers melted twice by the laser has less porosity and higher impact energy.

By summarizing the state of the art, it can be stated that the authors of all mentioned studies focus more on commonly used layer thicknesses of 20–50 µm, and therefore the area of higher layer thicknesses is rarely represented. This fact led the authors of this study to the idea of focusing on the creation of more productive parameters, which could then be transferred to a real production environment. However, the authors found the literature dealing with the same issue [8] (100 µm layer thickness), this was only at the level of single tracks, not volume samples [13,21], where the authors did not focus only on the constant layer thickness but addressed the range of 20–160 µm. From the analyzed studies, it can also be stated that this issue is very extensive because when optimizing the parameters, there are several unknowns in this process (laser power, scanning speed, etc.), which need to be chosen correctly. Poorly designed parameters, according to [19], may be insufficient to melt the metal powder, strong vaporization, and spatter, lead to voids and inclusions or leave some powders in an unmelted state. According to [10], this can lead to residual stresses and distortions, accompanying an increase in unwanted porosity. Therefore, it is appropriate to outline a methodology that could be used for the development and optimization of parameters for the far more efficient and productive production of parts using metallic 3D printing. This methodology based on multistage optimization of parameters for a specific layer thickness of 100 µm was designed and tested. The goal was to achieve a relative density of 99.9% in the volume samples produced, which was successfully met. This was verified by additional measurements using CT analysis. Furthermore, the best parameters were used for the production of samples for the tensile test, and this test was performed to analyze the mechanical properties of the parts thus produced.

## 2. Materials and Methods

### 2.1. Material and Production of Samples

Steel 1.2709 is a martensitic tool steel modified for hardening. Other international designations refer to it as 18Ni (300 grade) maraging steel, or X3NiCoMoTi 18-9-5. The material composition is shown in Table 1. This category of steel achieves its excellent mechanical properties due to the formation of low carbon martensite. This steel is characterized by excellent strength with high toughness and very good mechanical properties, which can be easily achieved by heat treatment options [22].

**Figure 1:**
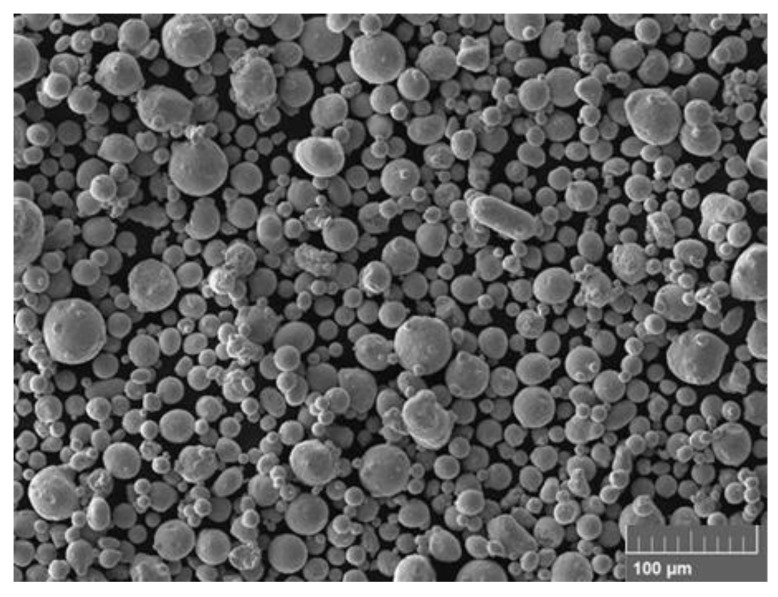
SEM (Scanning Electron Microscope) image of the material investigated.

The powder for the experiment used was MS1 from EOS GmbH (EOS GmbH—Electro Optical Systems, Krailling, Germany), and the samples were produced on an EOS M290 machine equipped with a 400 W laser in a nitrogen atmosphere. A basic analysis of the powder was performed on a CAMSIZER^®^ device (Hann, Germany), the particle mean size was 31.83 µm, and the average diameter of 32.10 µm was determined. For comparison, the powder manufacturer states a generic particle size distribution in the range of 20–65 µm. [24] For the powder analysis, the scanning electron microscope, Tescan Vega 3 LMU (TESCAN, Brno, Czech Republic), with accelerating voltage 20 kV, detector SE + BSE was used. Figure 1 shows the powder material by SEM (Scanning Electron Microscope). At first glance, there is a clear difference in the quality of individual particles, where irregular particles or satellites occur in the investigated powder, which could contribute to the imperfections of the internal structure.

### 2.2. Default and Recommended 3D Printing Parameters

Under standard circumstances, the parameters recommended and supplied by the manufacturer for the given material are used. According to the source [23], they are either EOSPRINT 1.0 Parameter Set MS1_Performance 1.0 with a layer thickness of 40 µm and a volume rate of 4.2 mm^3^/s or MS1_Speed 1.0 with a layer thickness of 50 µm and a volume rate of 5.5 mm^3^/s. This study aimed to create productive and functional parameters with a layer thickness of 100 µm to speed up the entire production process while maintaining the quality of the part. Due to the insufficient amount of work dealing with the 3D printing of tool steel 1.2709 at a layer thickness of 100 μm, it was necessary to further consider the information available for 3D printing at lower layers. The values obtained from this experiment, together with the others found for different layer thicknesses, are shown in Table 2, based on which the initial values for 3D printing optimization were built. The bold-highlighted parameters were not included in the calculation, as their volume energy is significantly different from the others.

The information provided by EOS GmbH (Krailling, Germany) served as another source of input information for the design of the process parameters. This information relates to the setting of parameters for the 3D printing of layers with a thickness of 20, 40, and 50 μm. The values are shown in Table 3.

The values obtained from the search section and EOS GmbH were then subjected to approximation. The approximations performed were quadratic, linear, and power. Using the obtained equations, the values of the process parameters were calculated. Based on the reliability coefficient R and the realistically achievable values, representative values were subsequently selected, which are listed in Table 4.

Based on the obtained values, the center of the process parameter matrix was determined for a power of 250 W and a scanning speed of 350 mm/s. Subsequently, the matrix of the proposed process parameters was compiled to contain the values obtained by approximating the search data as well as the values obtained by approximating the existing parameters. The proposed matrix of process parameters is shown in Table 5. These parameters were subsequently used to create single tracks.

### 2.3. Single Track Method and Sample Preparation

**Figure 2:**
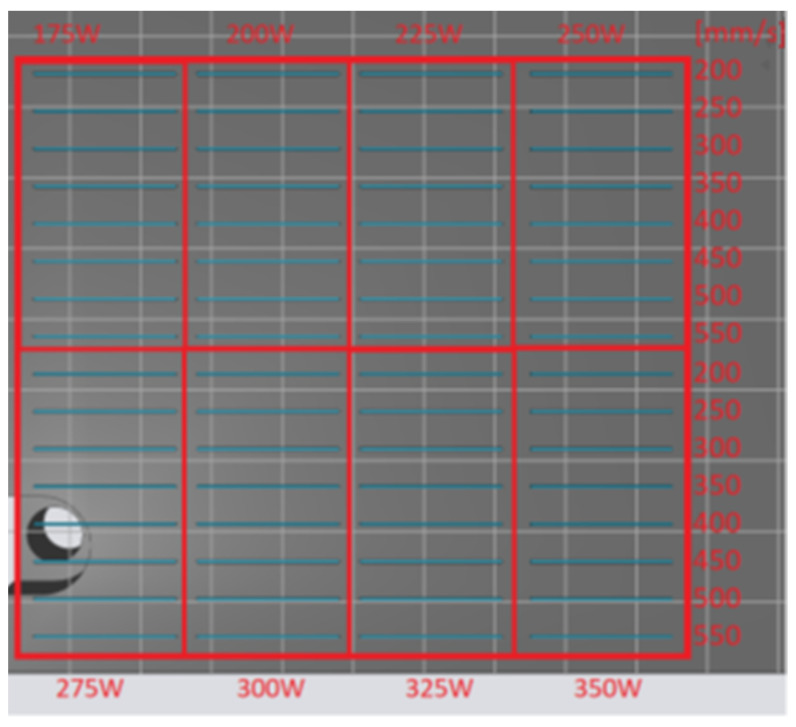
The layout of single tracks on the building platform.

Based on the proposed process parameters in Section 2.2., the construction of single tracks was performed. The individual single tracks were placed on the platform, as shown in Figure 2. The length of the individual single tracks was 20 mm.

**Figure 3:**
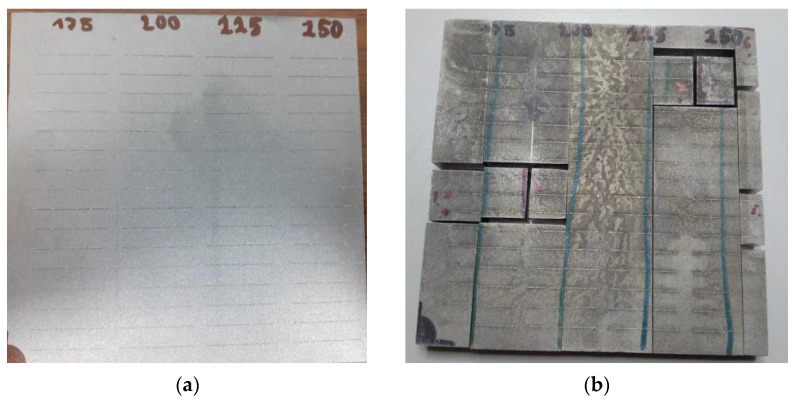
(**a**) Building platform with printed single tracks; (**b**) Platform cutting system with single tracks.

The building platform after 3D printing is shown in Figure 3a. It is possible to observe regular interruptions on individual welds (single tracks). This is not a defect caused during 3D printing or mechanical failure of the sample but a consequence of the 3D printing parameter, which affects the maximum length of the weld without interruption. In this case, it was 10 mm. In practice, this interruption is used due to a more even distribution of thermal stress of the printed object. For metallographic evaluation, it was necessary to cut the building platform. The first cuts were made by electric spark cutting. These sections are perpendicular to the welds so that they can be observed in the cross-section. A metallographic grinder could not be used to avoid thermal impact and deterioration of the samples. This was then used to reduce the samples to such a size that they could be pressed into a metallographic puck. Due to the close location of the welds, two to three samples fit into one puck, depending on the placement on the platform. The platform cutting system is shown in Figure 3b.

**Figure 4:**
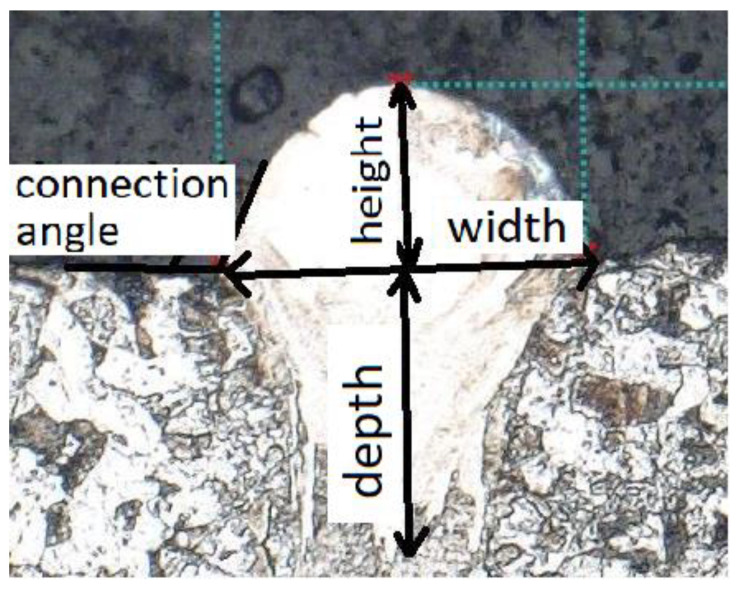
Observed parameters on the single track in this experiment (similar to [2]).

The samples were gradually pressed into the form of a metallographic puck. DuroFast molding compound from Struers was used, which is designed for the precise filling of fine edges. Pressing was followed by the polishing of the samples. Abrasive papers with a grain size of 120, 320, and 500 were used successively. This was followed by polishing with the addition of a diamond abrasive emulsion. It was first used with a particle size of 9 μm, then with 3 μm. To show the structure of the material, the samples were later etched with Nital (5% Nital solution (95 mL HCl + 5 mL HNO_3_). When evaluating the quality of the weld when looking at the section, it is advisable to monitor the depth of impact on the previous layer, and the weld height must be sufficient for optimal construction, the contact angle must be greater than 90° and must not contain cracks or other defects. For clarity, these parameters are shown in Figure 4 [29].

### 2.4. Preparation of Volume Samples—1st Step

For the next step of the optimization of the process parameters, after the implementation of single tracks, the 3D printing of volume samples was used. It is a matrix of cubic samples, each printed at different process parameters to find areas suitable for quality 3D printing.

Based on the results, Table 6 was compiled, which contains a proposal for combinations of individual parameters for volume samples. In total, four different axial distances (hatch distances) were varied on four construction platforms. The size of the individual volume bodies was 10 mm × 10 mm in the plan, so sixteen of them were placed on one construction platform of 80 mm × 80 mm, which corresponds to the design in Table 6. Thus, there were a total of 64 volume bodies.

Materialise Magics software was used to prepare the volume data. For better orientation and overview, the samples were marked with side numbering. The support system under volume samples was also configured here. After creating the volumetric data, the EOSPRINT software was used, in which the process parameters for the individual bodies were defined.

The preparation of the printed data was followed by the preparation of the 3D printing itself. It was necessary to clamp building platforms on the printer on which the build job will be carried out. The platforms had to be leveled so that the distance between the surface and the recoater was approximately the same at all points.

**Figure 5:**
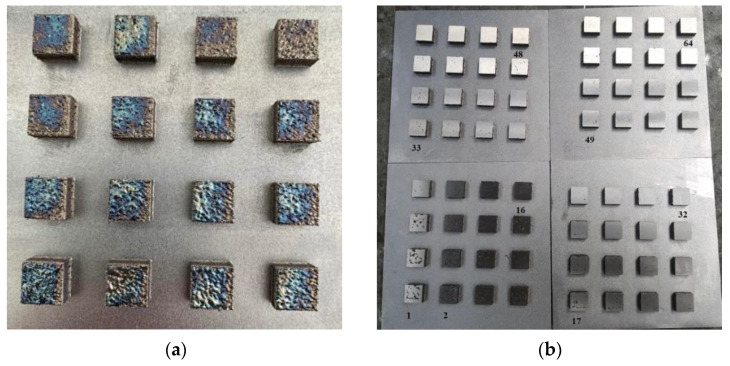
(**a**) Building job after 3D printing; (**b**) Building platforms after grinding.

After 3D printing and the cooling of the building platform, it was necessary to vacuum the excess powder material to avoid contamination of the working environment. The status of the completed print job is shown in Figure 5a. The condition of the surface layer indicates excessive volumetric energy, which can cause soot to form and the product to burn. To study the relative density, it was necessary to grind the surface layer. The condition of the pallets after grinding is visible in Figure 5b.

**Figure 6:**
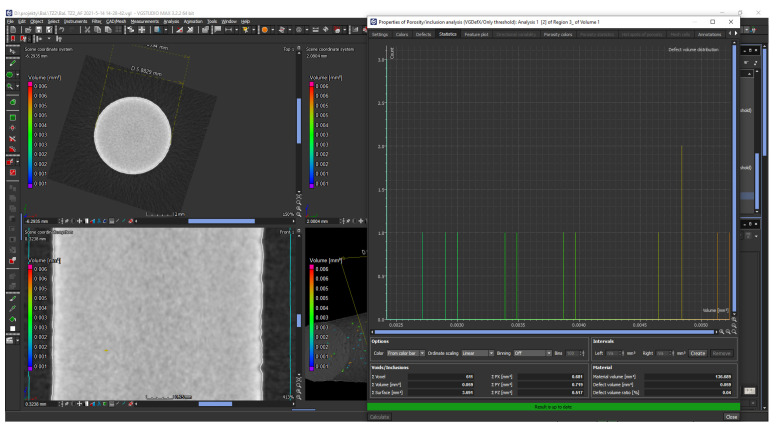
VGSTUDIO MAX 3.2 Porosity analysis of the sample.

Selected volume samples were mechanically separated from the building platform. A simple mechanical cut was proven to be the most effective. After separation, the samples were pressed and polished in the same way as described in Section 2.3. Except that the Multifast molding compound with Struers was used, and the samples were not etched. Due to the subsequent measurement of relative density, it was necessary to pay attention to the increased quality of the resulting surface. The light optical microscopy was carried out using a KEYENCE VHX-6000 (Osaka, Japan) connected to a personal computer with image analysis software (contamination analysis). The density of the manufactured components was estimated using an image analysis method to assess the porosity within a sample by measuring the percentage area of porosity on the polished surface. Furthermore, CT analysis of the porosity was performed for subsequent comparison. As in the study [30], a Zeiss Metrotom 1500 (Carl Zeiss AG, Oberkochen, Germany) equipped with an X-ray tube for a maximum of 225 kV acceleration voltage, 3000 A tube current, and 2 K detector resolution was used. The measurement was evaluated using VGSTUDIO MAX 3.2.2.152742 (Volume Graphics GmbH, Heidelberg, Germany), 64 bit and the Porosity/Inclusions Analysis module (Figure 6). The analysis was performed on six pieces of the evaluation part of the sample for the tensile test, where the results were used to verify the data obtained by measuring the porosity in metallographic samples.

The results of this step are shown in Section 3.2 and, at the same time, served to implement the next step.

### 2.5. Preparation of Volume Samples—Second Step

Based on the knowledge obtained from the first set of volume samples, a second set followed, the aim of which is to better focus on the area of potentially suitable process parameters. When designing the optimized matrix of process parameters, the conclusions obtained from the previous measurement were considered. The volume energy density should be up to 100 J/mm^3^, and the axial (hatch) distance should be higher than 0.095 mm. Furthermore, the efficiency of the process was considered, so the scanning speed was increased while maintaining the power of the laser to reduce the volume energy. The matrix of the proposed process parameters is shown in Table 7.

The 3D printing preparation procedure and the process of 3D printing volume samples were the same as described in the previous section. After 3D printing, it was evident that the failure to remove the powder material incidence of soot can result from excessive volume density of energy. This fact was confirmed by the results of the study [13] and was caused by the fact that the input heat energy cannot melt the metal powder. A better surface layer quality can be observed than with the first set of samples.

### 2.6. Tensile Test

**Figure 7:**
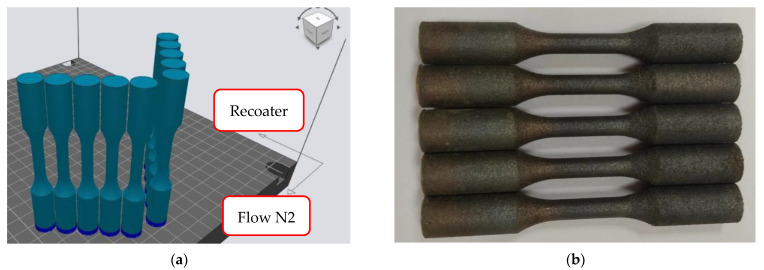
(**a**) Orientation of the samples in the printer workspace; (**b**) Printed test samples for tensile test.

This test was performed at 20 °C on a universal electromechanical testing machine of the LabTest model 5.100SP1 series according to the test standard EN ISO 6892-1 [31]. The evaluated characteristics were as follows: tensile strength [MPa], yield strength [MPa], and elongation at break [%]. Ten samples were printed to perform a tensile test to determine the mechanical properties of the optimized material. The orientation of the samples in the printer workspace is shown in Figure 7a. The resulting condition of the printed samples is shown in Figure 7b. The print was successful as the samples were without visible defects. Five test samples were left without heat treatment, and another five samples were precipitation hardened at 490 °C for 6 h, according to the manufacturer’s recommendations.

## 3. Results

### 3.1. Evaluation of Single Tracks

#### 3.1.1. Visual Evaluation

**Figure 8:**
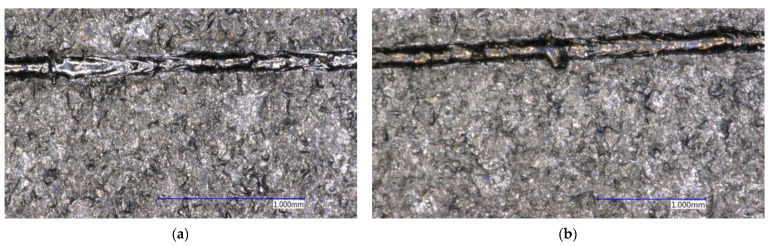
(**a**) Weld inhomogeneity P = 200 W, v = 550 mm/s; (**b**) Spatters of molten weld material P = 275 W, v = 275 mm/s.

Firstly, it was necessary to select single tracks by visual inspection. Macroscopic images were taken for this examination. The monitored welds must be continuous and free of visible defects, such as a missing part of the weld or the spatter of molten material. The already mentioned regular omission cannot be considered as a defect, as it is a definable parameter. An example of weld inhomogeneity is shown in Figure 8a, and an example of spatter is shown in Figure 8b.

**Figure 9:**
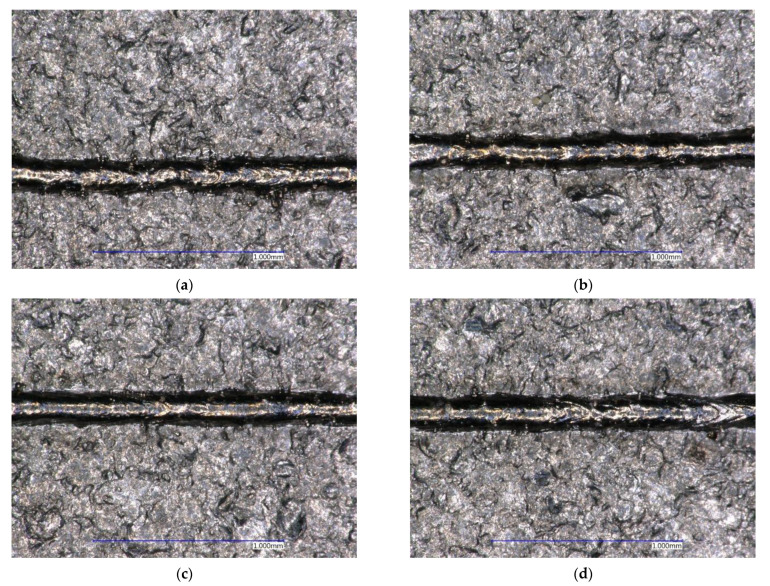
Visually suitable single tracks (**a**) P = 225 W, v = 200 mm/s; (**b**) P = 250 W, v = 200 mm/s; (**c**) P = 275 W, v = 250 mm/s; (**d**) P = 300 W, v = 250 mm/s.

Based on a visual inspection, potentially suitable samples were selected and shown in green in Table 8. A view of these individual samples is shown in Figure 9. The samples marked in yellow contained a certain defect, which would not have to appear during the volume build job. The samples marked in red contained undesirable defects, so they were evaluated as unsuitable within single tracks.

#### 3.1.2. Evaluation Based on Metallographic Analysis

**Figure 10:**
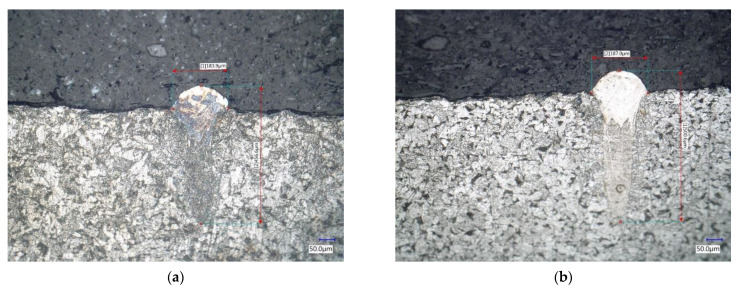

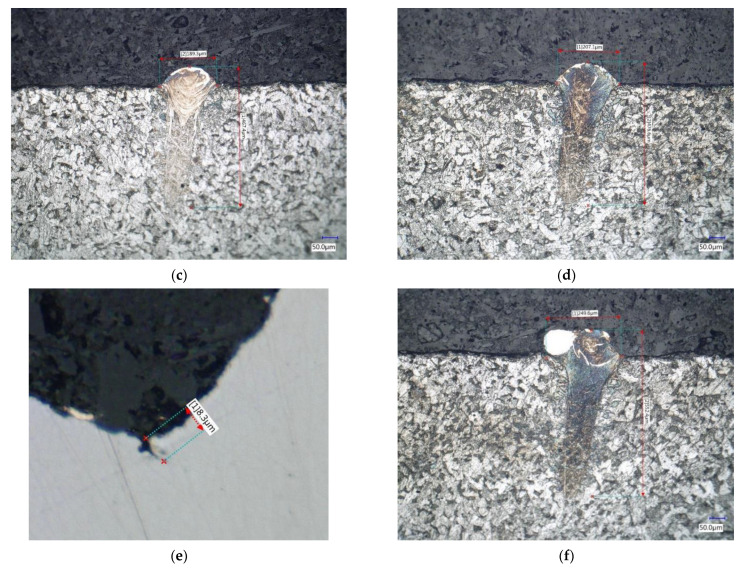
Metallographic images of selected welds (**a**) weld No. 3; (**b**) weld No. 6; (**c**) weld No. 10; (**d**) weld No. 12; (**e**) crack on weld No. 7; (**f**) defect on weld No. 11.

Based on the measurements, Table 9 was compiled, which contains the measured monitored parameters for the selected welds. Welds No. 3, 6, 10, and 12 marked in green are OK. The yellow-marked welds No. 4, 5, and 8 are in order from the point of view of metallographic examination, but during the visual examination of the entire weld metal, certain small defects were revealed. Welds marked in red were evaluated as unsuitable. Welds No. 1 and 2 are unsuitable due to insufficient metallurgical connection with the substrate. A crack was found in weld No. 9 at the substrate-weld interface, which is shown in Figure 10a. Weld No. 11 was discarded due to finding a defect at the cut point shown in Figure 10b. Welds No. 13, 14, and 15 were evaluated as too low.

The gray marked weld No. 7 should be evaluated as unsuitable, as it contains cracks between the weld and the substrate. The cracks found are shown in Figure 10e,f. Due to the probable placement of the section plane of the metallographic cut directly into the already mentioned regular omission, it is not possible to unambiguously exclude this weld. A view of the selected metallographic images of welds is shown in Figure 10.

Based on the examination of individual welds, the welds printed with the parameters shown in Table 10 were selected. Based on these findings, a suitable area, E_2_, appears to be in the range of 11–12.5 J/mm^2^. Based on the results, single tracks with a higher value of laser power were better evaluated. One of the perspective parameters confirms the results obtained from [8], P = 250 W, v = 200 mm/s. The authors agree with the statement from [18] that the promising area of laser power was in the range of 250–350 W. However, due to the higher layer thickness, it was necessary to reduce the scanning speed, which in this case appears to be optimal in the range of 200–300 mm/s. At the same time, the authors agree with the results from [19] that high laser power and low scanning speed lead to voids and inclusions.

The findings were used in the design of a matrix of process parameters for 3D printing volume samples.

### 3.2. Evaluation of Volume Samples–First Step

#### 3.2.1. Visual Evaluation

After grinding the surface layers, visual evaluation could begin. Samples with a high volume energy density were the worst. These are always samples in the corner (the farthest side from the recoater) of each building platform. This can be seen from the platform containing samples No. 1 to 16. Furthermore, it is possible to observe the difference between the individual platforms, which differ in the axial distance. With increasing axial distance, the samples are less porous at first glance. Based on visual inspection, potentially suitable volume samples were selected. These are samples that, at first sight, did not have visible porosity. These are samples 16, 45, 46, 47, 63, and 64.

#### 3.2.2. Evaluation Based on Metallographic Analysis

**Figure 11:**
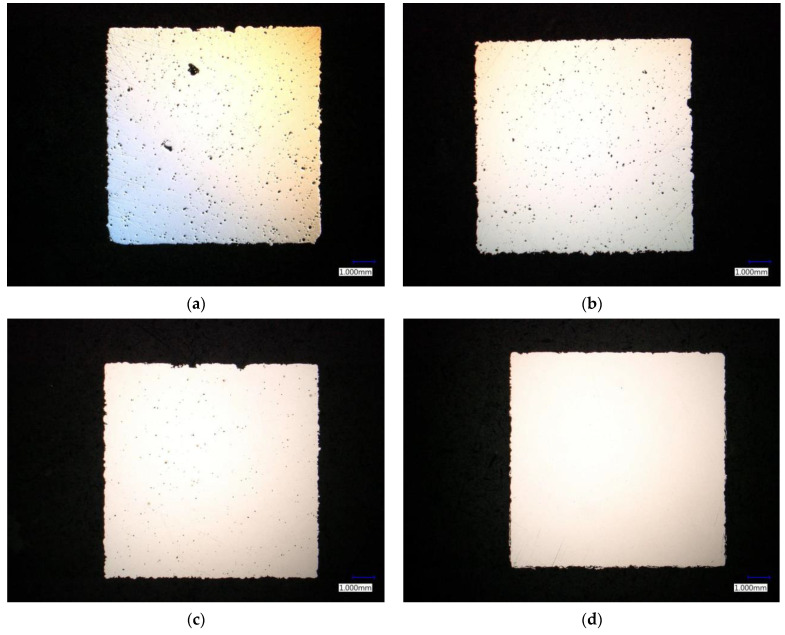
Images of metallographic sections (**a**) sample No. 46 (1st step); (**b**) sample No. 64 (1st step); (**c**) sample No. 15 (2nd step); (**d**) sample No. 64 (2nd step).

Images of individual sections were taken with a microscope. Figure 11a is an image of sample No. 46, where larger pores appeared after grinding. Figure 11b shows an image of sample No. 64, whose relative density was measured to be 99.319%. As further shown in Table 11, the resulting relative density of the selected samples did not differ much.

According to [32], the defects arising on the samples are caused by an excessive density of volume energy. This statement corresponds to the observed dependence on the samples, where the relative density increases with decreasing volume energy density. In contrast to the conclusions found for single tracks, welds appear to be suitable, which are printed with a lower density of volume energy. This difference may be due to a different method of warming up and not requiring as much energy for a perfect metallurgical bond as a single track. The study’s claim [3] that it is common for steel to stay within the range of volume energy of 50–100 J/mm^3^ can also be partially refuted. Even above the value of 100 J/mm^3^, acceptable results of relative density up to 99% can be achieved, provided that the correct combination of other parameters is observed. Thus, for another set of volume samples, an attempt was made to direct the volume energy density to a maximum value of 100 J/mm^3^ to reach a relative density of 99.9% and better.

### 3.3. Evaluation of Volume Samples–Second Step

#### 3.3.1. Visual Evaluation

For visual evaluation, it was necessary to remove the surface layer again. It is possible to observe much smaller area samples with an eye-apparent porosity.

Based on visual inspection, volume samples with a visible surface defect were discarded. Furthermore, the samples were examined and discarded based on a side view, where the condition of the surface and marking numbers were evaluated. A total of 12 volume samples were selected, with an emphasis on process efficiency, which was further modified into a metallographic cut. Samples Nos. 15, 16, 28, 32, 44, 47, 48, 56, 59, 60, 63 and 64 were selected.

#### 3.3.2. Evaluation Based on Metallographic Analysis

The prepared metallographic sections were subjected to analysis using a microscope. Due to the small size of the pores, gradual scanning of the cut surface at 100 times magnification was performed. After photographing the surface, a porosity analysis was performed. Figure 11c shows an image of sample No. 15, for which a relative density of 99.644% was measured. Compared to sample No. 64 shown in Figure 11d, for which the measured density was 99.982%, the difference is significant.

The results of this part are shown in Table 12. Based on the measured values of the second volume samples, it appears as a prospective area with a volume energy, E_3_, of 60–70 J/mm^3^, laser power, P, 300–350 W, scan speed around 500 mm/s, and an axial (hatching) distance near 0.11 mm. The process parameters of sample No. 64, marked in bold, were selected for 3D printing and the test samples for tensile tests with relative density 99.982%.

After the next step of optimization, the authors concluded that values above 100 J/mm^3^ are applicable, but above this value, it is not possible to achieve the required quality of parts with a relative density of 99.9%. Therefore, the conclusion from [3] that it is better to keep in the range of 50–100 J/mm^3^ can be confirmed. To achieve the recommended values of E_3_, it was necessary to increase v to the range of 440–500 mm/s and h to around 0.11 mm.

**Figure 12:**
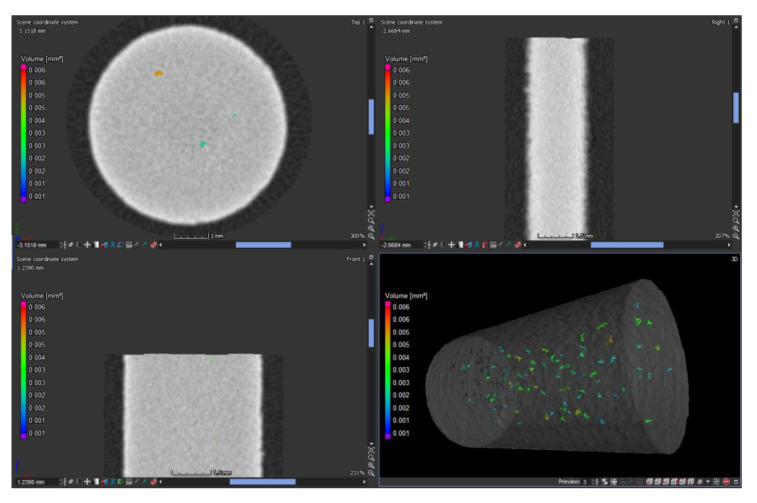
VGSTUDIO MAX 3.2 porosity analysis of the sample.

Subsequently, a porosity analysis was performed on the cylindrical parts of the manufactured bodies for the tensile test to compare the values obtained from the analysis of metallographic cuts. Porosity has been evaluated by computed tomography (Figure 12), and average parameters were calculated from the analysis of 6 samples (Table 13). The average porosity was 99.97%, the maximum porosity was 99.98%, and the minimum porosity reached 99.96%. This confirmed the value obtained from the evaluation of metallographic cuts of volume samples. Visualization of CT results also showed an even distribution of porosity over the entire volume sample without local agglomeration.

### 3.4. Tensile Test Results

The implementation of the tensile test was used to compare the basic mechanical properties with the obtained parameters for 100 µm and standard parameters from the company EOS for commonly used layer thicknesses. As it is a martensitic tool steel, the results correspond to this. The high values of the tensile strength limit for all test bars exceed the limit of 900 MPa in the phase after 3D printing and 1500 MPa after heat treatment. The biggest noticeable difference between the above groups of materials is recorded by the ductility characteristics. From the results of the elongation at the break, the produced material with the applied layer thickness of 100 µm is very brittle, and the value of elongation is not even 1%. If the authors compare it with the values declared by the manufacturer, it can be seen as 5 to 10 times lower (Table 14). This fact can lead, especially in the case of HPDC tools, to a brittle failure of these tools due to the low toughness of the material. It would also be appropriate to support this fact by examining the dynamic properties of a given material from the point of view of fatigue and comparing them with materials made with commonly available parameters. The results of this study confirm the conclusion [21] that the increase in the layer thickness decreases the elongation at the brake. However, at the same time, the partial conclusion of [21] did not confirm that strength values are not affected, as significant differences were measured in the tensile test sample. For samples without heat treatment, the differences in mechanical properties were in the range of 8–17% for heat-treated samples, these differences were more pronounced in the range of 8–21%.

**Figure 13:**
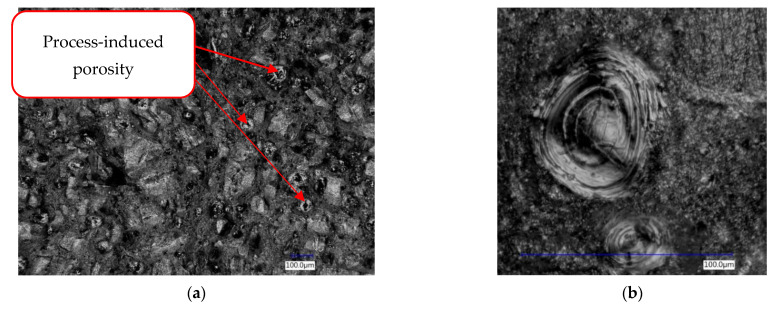
Fracture analysis with the Keyence VHX-6000 Digital Microscope (**a**) Fracture surface; (**b**) Discovered pores on the surface, close-up view.

Within all fracture surfaces, porosity is detectable (Figure 13a). Since this does not affect the tensile test and its results dramatically, for a fatigue test, this level of porosity would be considerable. Pores exposed within a fracture surface area range from 20 µm in cross-section diameter up to 100 µm (Figure 13b). It is important to know that the failure surface is very rugged. Within the surface, a mixed fracture feature with shear areas and porosity may be observed. For all samples, it was possible to observe heat cracks from a laser-induced melting process, which may be possible initiators of fatigue cracks and their development.

## 4. Discussion

In this study, the proposed methodology for the optimization of process parameters for additively produced tool steel 1.2709 for production with a layer thickness of 100 µm was investigated. According to the authors, this is one of the ways to increase the productivity of a given process while rapidly reducing production costs.

The principles, methodologies, and experiments of other authors in the given issue were analyzed in a relatively extensive state of the art part. The main shortcoming was the absence of publications describing the use of higher layer thickness, especially in the already mentioned 100 µm. This is mainly because the other authors focused mainly on the area of the commonly used layer thickness (20−50 µm), as this is already a proven area. Studies concerning the thickness of 100 µm were discovered, but, for example, in [8], the authors solved only single tracks, not solid samples, or in [13,21], the authors did not solve the constant layer thickness, but the range 20–160 µm. The researched issue is extensive and interconnected, as there are several parameters in the process that need to be chosen correctly. Their bad selection leads to, for example, according to [19], the impossibility of melting the metal powder, strong vaporization and spatter, creating voids and inclusions or leaving some powders in an unmelted state, or according to [10], it can lead to residual stresses, distortions, and unwanted porosity. Therefore, it was appropriate to design a methodology that will serve for the development and optimization of parameters for more efficient and productive production of parts using metallic 3D printing. This methodology, based on several stages of parameter optimization for a layer thickness of 100 µm, was designed and tested.

In the first step, it was necessary to make a default set of values. Based on the obtained values from the state of the art and parameters from EOS GmbH, the center of the process parameters complex was determined for a laser power of 250 W and a scanning speed of 350 mm/s. Subsequently, the combination of the proposed process parameters was compiled to contain the values obtained by approximating the research data as well as the values obtained by approximating the existing parameters. These parameters were subsequently used to create single tracks.

Based on the examination of individual welds in the single track phase, it was found that the area energy, E_2_, values in the range of 11–12.5 J/mm^2^ appeared to be a suitable area for further investigation. Furthermore, these values were used for the two-step optimization of parameters on volume samples. Based on the achieved results, single tracks with a laser power in the range of 250–350 W were better evaluated, which is the result from [18]. However, due to the higher layer thickness, it was necessary to reduce the scanning speed value, which for single tracks was in the range of 200–300 mm/s. One of the perspective parameters came out the same as in [8] with P = 250 W and v = 200 mm/s. At the same time, in this step, it can be agreed that high laser power and low scanning speed lead to voids and inclusions that also occurred in the samples [19].

After the first step of volume samples, the theory was confirmed with the formation of defects on the samples. According to the work [32], this was caused by the excessive density of volume energy. This statement correlates with the observed dependence on samples, where the relative density increases with decreasing volume energy density. During the evaluation of the experiments, the fact was confirmed that too much energy also causes visual defects. These samples were considered unsatisfactory. It can be expected if this phenomenon occurs while 3D printing real parts, most places that are visually poor, will not reach the required quality parameters. This confirms the result of a study [13] that a poorly designed input heat energy cannot melt the metal powder. Thus, for the second set of volume samples, an attempt was made to direct the volume energy density to a maximum value of 100 J/mm^3^. In contrast to the conclusions found for single tracks, the welds appear to be visually suitable, which are printed with a lower density of volume energy. This difference may be due to a different way of warming up and not requiring as much energy for a perfect metallurgical connection as a single track. The best results, after the first step, were achieved with a relative density of 99.319%, which can be considered an acceptable result, but the authors sought to achieve a relative density of at least 99.9%. This can partially refute the statement of the study [3] because even above the value of 100 J/mm^3^, acceptable results of relative density, around 99%, can be achieved, provided that the correct combination of other parameters is observed.

After the second step, it can be stated that based on the measured values of the second volume samples, the following is the perspective area for the given material and the layer thickness of 100 µm:

- Volume energy density E_3_ 60–70 J/mm^3^,
- Laser power P 300–350 W,
- Scanning speed v around 500 mm/s,
- Hatching distance h near 0.11 mm.

The best result, relative density 99.982%, was obtained by sample 64, which was produced with the 3D printing parameters P = 340 W, v = 500 mm/s, h = 0.11 mm, and E_3_ = 61.8 J/mm^3^. To refine the study, the relative density value was verified by CT analysis of the produced samples for the tensile test and confirmed the results obtained from the analysis of metallographic cuts. The measured value was in the range of 99.97 ± 0.01%.

This experiment proved that all these process parameters are interrelated, and it is necessary to follow the recommended ratio for E_3_. This can be seen from the differences in the results of the analysis of volume samples, when the relative change of the parameter v (from 300 to 500 mm/s) and the preservation of the parameters P and h reached a relative density of up to 99.98%. Although this study is conceptually similar to the sources [8,9,10,12,13,14,18,19], the results are not comparable, mainly due to the layer thickness parameter, which the authors chose in the commonly used range of 20–50 µm. However, based on the obtained results from the performed experiments, it is possible to confirm the dependence from [19] at the layer thickness 100 µm, that low laser power and high scanning speed were insufficient to melt the metal powder. However, high laser power and low scanning speed will bring strong vaporization and spatter, which leads to voids and inclusions.

This result can be considered successful because the use of these parameters in potential applications will reduce the cost of 3D printing for HPDC tools by up to 40%.

Another point in the discussion is the final position of the samples with the smallest porosity. Here, the results of a study by Herbold et al. [33] confirm that the powder bed porosity could be found to increase along the recoating direction (the farthest from the powder dispenser). In addition to this, the spatter from the previous scanned layer would be dragged towards the front of the build platform as the new layer of powder is being spread. These will contribute to additional porosity in the parts located in the front of the build platform. In this experiment, this was confirmed, as the best quality was achieved by the samples that were placed in the upper right corner (closest to the recoater). This fact is also confirmed by the practical experience recommended by users of metal 3D printers, namely, to place parts as close as possible to the recoater.

From the results of the tensile test, the produced material with the applied layer thickness of 100 µm is very brittle, and the value of elongation is not even 1%. If the authors compare the elongation values with the values declared by the manufacturer, values from the experiment were 5 to 10 times lower. This fact can lead, especially in the case of HPDC tools, to a brittle failure of these tools due to the low toughness of the material. It would also be appropriate to support this fact by examining the dynamic properties of a given material from the point of view of fatigue and comparing them with materials made with commonly available parameters. Because the most important thing is, as mentioned in article [34,35], the connection of optimal mechanical properties with precise functional surfaces. Compared to the study [21], the conclusion agrees that the increase in the layer thickness decreases the elongation at the break, but it is not agreed with the partial conclusion that the strength values are not affected, as the samples without heat treatment decreased the mechanical properties in the range of 8–17% and heat-treated samples to be reduced by up to 8–21%.

Regarding the results, it would be appropriate to perform a third iteration of volume samples, which would have an input energy in the range of 55–65 J/mm^3^. From the observed results, it would be possible to expect a relative porosity of 99.99%.

## Figures and Tables

**Table 1:** The chemical composition of MS1 material [23].

Powder Chemical Composition (w.t. %)	Fe	Ni	Co	Mo	Ti	Al	Cr, Cu	Mn, Si	P, S
Max	Balance	19	9.50	5.20	0.80	0.15	0.5	0.1	0.01
Min		17	8.50	4.50	0.60	0.05	-	-	-

**Table 2:** Parameters obtained based on the state of the art.

P (W)	v (mm/s)	t (mm)	h (mm)	E_1_ (J/mm)	E_2_ (J/mm^2^)	E_3_ (J/mm^3^)	Source
250	250	0.1	0.15	1.00	10.00	66.66	[8]
300	700	0.05	0.12	0.43	8.57	71.43	[22]
285	960	0.04	0.11	0.30	7.42	67.47	[25]
258	960	0.04	-	0.27	6.72	-	[26]
275	1091	0.03	0.12	0.25	8.40	70	[27]
90	220	0.03	0.14	0.41	13.64	97.4	[28]
**100**	**180**	**0.03**	**0.14**	**0.56**	**18.52**	**132.28**	**[27]**
**105**	**150**	**0.03**	**0.125**	**0.70**	**23.33**	**186.67**	**[28]**
**200**	**340**	**0.03**	**0.1**	**0.59**	**19.61**	**196.08**	**[9]**

**Table 3:** Table of process parameters by EOS GmbH.

t (mm)	P (W)	v (mm/s)	E_3_ (J/mm^3^)
0.02	150	800	93.75
0.04	285	960	62.47
0.05	305	1010	54.91

**Table 4:** Values of process parameters approximation.

Values from	t (mm)	P (W)	V (mm/s)	E_2_ (J/mm^2^)
State of the art	0.1	249	242	10.29
EOS GmbH	0.1	281	480	5.85

**Table 5:** Proposed matrix of process parameters (area energy density E_2_ (J/mm^2^)).

P (W]	v 200 (mm/s)	v 250 (mm/s)	v 300 (mm/s)	v 350 (mm/s)	v 400 (mm/s)	v 450 (mm/s)	v 500 (mm/s)	v 550 (mm/s)
175	8.75	7.00	5.83	5.00	4.38	3.89	3.50	3.18
200	10.00	8.00	6.67	5.71	5.00	4.44	4.00	3.64
225	11.25	9.00	7.50	6.43	5.63	5.00	4.50	4.09
250	12.50	10.00	8.33	7.14	6.25	5.56	5.00	4.55
275	13.75	11.00	9.17	7.86	6.88	6.11	5.50	5.00
300	15.00	12.00	10.00	8.57	7.50	6.67	6.00	5.45
325	16.25	13.00	10.83	9.29	8.13	7.22	6.50	5.91
350	17.50	14.00	11.67	10.00	8.75	7.78	7.00	6.36

**Table 6:** Proposed parameters for 3D printing volume samples (volume energy density E_3_ (J/mm^3^)).

P [W]	v 200 (mm/s)	v 225 (mm/s)	v 250 (mm/s)	v 275 (mm/s)	v 300 (mm/s)
225 (h = 0.095 mm)	118.4	105.3	94.7	-	78.9
250	131.6	117.0	105.3	-	87.7
275	-	128.7	115.8	105.3	-
300	-	140.4	126.3	114.8	105.3
325	-	-	-	-	114.0
225 (h = 0.1 mm)	112.5	100.0	90.0	-	75.0
250	125.0	111.1	100.0	-	83.3
275	-	122.2	110.0	100.0	-
300	-	133.3	120.0	109.1	100.0
325	-	-	-	-	108.3
225 (h = 0.105 mm)	107.1	95.2	85.7	-	71.4
250	119.0	105.8	95.2	-	79.4
275	-	116.4	104.8	95.2	-
300	-	127.0	114.3	103.9	95.2
325	-	-	-	-	103.2
225 (h = 0.11 mm)	102.3	90.9	81.8	-	68.2
250	113.6	101.0	90.9	-	75.8
275	-	111.1	100.0	90.9	-
300	-	121.2	109.1	99.2	90.9
325	-	-	-	-	98.5

**Table 7:** Design of a matrix of process parameters after considering the results from the previous step.

Sample	P (W)	v (mm/s)	h (mm)	E_3_ (J/mm^3^)	Sample	P (W)	v (mm/s)	h (mm)	E_3_ (J/mm^3^)
1	280	300	0.1	93.9	33	320	300	0.1	106.7
2	280	370	0.1	75.7	34	320	370	0.1	86.5
3	280	440	0.1	63.6	35	320	440	0.1	72.7
4	280	500	0.1	56.0	36	320	500	0.1	64.0
5	280	300	0.103	90.6	37	320	300	0.103	103.6
6	280	370	0.103	73.5	38	320	370	0.103	84.0
7	280	440	0.103	61.8	39	320	440	0.103	70.6
8	280	500	0.103	54.4	40	320	500	0.103	62.1
9	280	300	0.106	88.1	41	320	300	0.106	100.6
10	280	370	0.106	71.4	42	320	370	0.106	81.6
11	280	440	0.106	60.0	43	320	440	0.106	68.6
12	280	500	0.106	52.8	44	320	500	0.106	60.4
13	280	300	0.11	84.8	45	320	300	0.11	97.0
14	280	370	0.11	68.8	46	320	370	0.11	78.6
15	280	440	0.11	57.9	47	320	440	0.11	66.1
16	280	500	0.11	50.9	48	320	500	0.11	58.2
17	300	300	0.1	100.0	49	340	300	0.1	113.3
18	300	370	0.1	81.1	50	340	370	0.1	91.9
19	300	440	0.1	68.2	51	340	440	0.1	77.3
20	300	500	0.1	60.0	52	340	500	0.1	68.0
21	300	300	0.103	97.1	53	340	300	0.103	110.0
22	300	370	0.103	78.7	54	340	370	0.103	89.2
23	300	440	0.103	66.2	55	340	440	0.103	75.0
24	300	500	0.103	58.3	56	340	500	0.103	66.0
25	300	300	0.106	94.3	57	340	300	0.106	106.9
26	300	370	0.106	76.5	58	340	370	0.106	86.7
27	300	440	0.106	64.3	59	340	440	0.106	72.9
28	300	500	0.106	56.6	60	340	500	0.106	64.2
29	300	300	0.11	90.9	61	340	300	0.11	103.0
30	300	370	0.11	73.7	62	340	370	0.11	83.5
31	300	440	0.11	62.0	63	340	440	0.11	70.2
32	300	500	0.11	54.5	64	340	500	0.11	61.8

**Table 8:** Evaluation of single tracks based on visual inspection (red—unsuitable, yellow—partially suitable, green—suitable).

P (W)	v 200 (mm/s)	v 250 (mm/s)	v 300 (mm/s)	v 350 (mm/s)	v 400 (mm/s)	v 450 (mm/s)	v 500 (mm/s)	v 550 (mm/s)
175	8.75	7.00	5.83	5.00	4.38	3.89	3.50	3.18
200	10.00	8.00	6.67	5.71	5.00	4.44	4.00	3.64
225	11.25	9.00	7.50	6.43	5.63	5.00	4.50	4.09
250	12.50	10.00	8.33	7.14	6.25	5.56	5.00	4.55
275	13.75	11.00	9.17	7.86	6.88	6.11	5.50	5.00
300	15.00	12.00	10.00	8.57	7.50	6.67	6.00	5.45
325	16.25	13.00	10.83	9.29	8.13	7.22	6.50	5.91
350	17.50	14.00	11.67	10.00	8.75	7.78	7.00	6.36

**Table 9:** Evaluation of selected welds based on metallographic analysis (red + grey—unsuitable, yellow—partially suitable, green—suitable).

Weld	P (W)	v (mm/s)	Width (µm)	Height (µm)	Depth (µm)
1	175	550	153	54	92
2	200	550	134	38	109
3	225	200	184	74	111
4	225	250	145	73	119
5	225	300	152	67	122
6	250	200	178	66	124
7	250	250	166	62	128
8	250	300	163	77	113
9	275	200	204	79	129
10	275	250	179	62	144
11	300	200	230	77	161
12	300	250	214	63	184
13	350	200	170	46	201
14	350	250	189	24	202
15	350	300	195	32	167

**Table 10:** The best-evaluated welds.

Weld	P (W)	v (mm/s)
3	225	200
6	250	200
10	275	250
12	300	250

**Table 11:** The resulting relative density of selected samples after the 1st step.

Sample	Rel. Density (%)	P (W)	v (mm/s)	h (mm)	E_3_ (J/mm^3^)
16	99.127	325	300	0.095	114
45	99.059	300	250	0.105	114.3
46	98.91	300	275	0.105	103.9
47	99.094	300	300	0.105	95.2
63	98.931	300	300	0.11	90.9
64	99.319	325	300	0.11	98.5

**Table 12:** The resulting relative density of selected samples after the 2nd step.

Sample	Rel. Density (%)	P (W)	v (mm/s)	h (mm)	E_3_ (J/mm^3^)
15	99.644	280	440	0.11	57.9
16	99.8	280	500	0.11	50.9
28	99.811	300	500	0.106	56.6
32	99.861	300	500	0.11	54.5
44	99.761	320	500	0.106	60.4
47	99.582	320	440	0.11	66.1
48	99.968	320	500	0.11	58.2
56	99.977	340	500	0.103	66.0
59	99.959	340	440	0.106	72.9
60	99.973	340	500	0.106	64.2
63	99.96	340	440	0.11	70.2
**64**	**99.982**	**340**	**500**	**0.11**	**61.8**

**Table 13:** The resulting relative density of 3D printed samples after CT analysis.

Sample	Relative Density (%)
1	99.97
2	99.98
3	99.96
4	99.98
5	99.98
6	99.96

**Table 14:** Tensile test results and comparison with values declared by EOS.

Sample	Tensile Strength (MPa)	Yield Strength (MPa)	Elongation at Break (%)
100 µm HT	1775 ± 18	1528 ± 16	0.7 ± 0.3
100 µm AB	997 ± 8	945 ± 10	0.8 ± 0.8
EOS 1.2709 40 µm HT	2260	2180	3.3
EOS MS 1 40 µm AB 400 W	1100 ± 150	930 ± 150	-
EOS MS 1 40 µm HT 200 W	2080	2000	4
EOS MS 1 50 µm AB M 400	1200	1070	11
EOS MS 1 50 µm HT M 400	2080 ± 100	2030 ± 100	2 ± 1

## Data Availability

Data is contained within the article.
